# Neuroimaging of posttraumatic stress disorder in adults and youth: progress over the last decade on three leading questions of the field

**DOI:** 10.1038/s41380-024-02558-w

**Published:** 2024-04-17

**Authors:** Cecilia A. Hinojosa, Grace C. George, Ziv Ben-Zion

**Affiliations:** 1 Department of Psychiatry and Behavioral Sciences, Emory University School of Medicine, Atlanta, GA USA; 2 Department of Psychiatry, McLean Hospital, Belmont, MA USA; 3 Department of Comparative Medicine, Yale University School of Medicine, New Haven, CT USA; 4 Department of Psychiatry, Yale University School of Medicine, New Haven, CT USA; 5 US Department of Veterans Affairs National Center for PTSD, VA Connecticut Healthcare System, West Haven, CT USA

**Keywords:** Neuroscience, Diagnostic markers

## Abstract

Almost three decades have passed since the first posttraumatic stress disorder (PTSD) neuroimaging study was published. Since then, the field of clinical neuroscience has made advancements in understanding the neural correlates of PTSD to create more efficacious treatment strategies. While gold-standard psychotherapy options are available, many patients do not respond to them, prematurely drop out, or never initiate treatment. Therefore, elucidating the neurobiological mechanisms that define the disorder can help guide clinician decision-making and develop individualized mechanisms-based treatment options. To this end, this narrative review highlights progress made in the last decade in adult and youth samples on three outstanding questions in PTSD research: (1) Which neural alterations serve as predisposing (pre-exposure) risk factors for PTSD development, and which are acquired (post-exposure) alterations? (2) Which neural alterations can predict treatment outcomes and define clinical improvement? and (3) Can neuroimaging measures be used to define brain-based biotypes of PTSD? While the studies highlighted in this review have made progress in answering the three questions, the field still has much to do before implementing these findings into clinical practice. Overall, to better answer these questions, we suggest that future neuroimaging studies of PTSD should (A) utilize prospective longitudinal designs, collecting brain measures before experiencing trauma and at multiple follow-up time points post-trauma, taking advantage of multi-site collaborations/consortiums; (B) collect two scans to explore changes in brain alterations from pre-to-post treatment and compare changes in neural activation between treatment groups, including longitudinal follow up assessments; and (C) replicate brain-based biotypes of PTSD. By synthesizing recent findings, this narrative review will pave the way for personalized treatment approaches grounded in neurobiological evidence.

## Introduction

In 2013, the American Psychiatric Association revised the posttraumatic stress disorder (PTSD) criteria in the 5^th^ edition of its “Diagnostic Statistical Manual of Mental Disorders” (DSM-5). While the DSM-IV classified PTSD under “Anxiety Disorders,” the DSM-5 has repositioned it within a newly established category of “Trauma-and Stressor-Related Disorders.” According to the DSM-5, traumatic events are defined as exposure to actual or threatened death, serious injury, or a threat to the physical integrity of oneself or others, either directly (witnessing trauma) or indirectly (learning that trauma happened to a close relative or friend) [1]. In addition to trauma exposure (i.e., criterion A), four symptom clusters that characterize the disorder are persistent re-experiencing the trauma (i.e., criterion B), avoiding people, places, or thoughts related to the trauma (i.e., criterion C), negative thoughts and feelings that began or worsened after the trauma (i.e., criterion D); and trauma-related increased in arousal and reactivity (i.e., criterion E). Symptoms must last at least one month, not be caused by drugs or other illnesses, and cause significant functional impairment. The DSM-5 introduced a developmental subtype of PTSD specifically for children aged six years or younger, aligning closely with adult diagnostic criteria. However, it adapts criterion A for this age group, allowing for indirect exposure to trauma, such as through witnessing an event or learning about a traumatic event affecting a parent or caregiver [1].

## Epidemiology and prognosis

Worldwide, up to 70% of the adult population will experience at least one traumatic event (as defined by criterion A) in their lifetime [2], and the prevalence of PTSD ranges from 2 to 9% [3]. Indeed, four post-traumatic symptom trajectories have been highlighted in literature: resiliency, recovery, chronic, and delayed onset, the most common being the resiliency trajectory [4, 5]. Similarly, childhood trauma exposure is common, with up to two-thirds of youth reported having experienced a traumatic event and almost 5% of trauma-exposed youth meeting the criteria for PTSD [6–8]. It is important to note that females are more prone to developing PTSD than males [9, 10]. This disparity is theorized to be attributed to trauma one is exposed to, with females experiencing more interpersonal violence than males [11]. However, even when controlling for trauma type, females still exhibit greater PTSD prevalence [11]. This suggests that biological factors may contribute to this disparity. Given this, more studies are introducing sex as a biological variable to explore this disparity further. For in-depth reviews, see [12, 13].

PTSD is a debilitating disorder in many aspects across the lifespan. A PTSD diagnosis contributes to billions in annual productivity loss [14] and increased medical problems [15] and is associated with a variety of co-occurring disorders, including substance use disorders [16], depression, and anxiety [17]. For individuals diagnosed with PTSD, many treatment options are available. The most empirically supported options are trauma-focused interventions [18]. Of psychotherapy options available, up to half of the patients who complete treatment will show clinically meaningful improvement [19–21], and many patients prematurely drop out of treatment before receiving an adequate dose [22]. Furthermore, many patients fail to seek treatment altogether, especially in marginalized groups [23]. Given the considerable overlap between PTSD, depression, and anxiety, and because research still has not identified pharmaceutical targets specific for PTSD, sertraline and paroxetine are FDA-approved pharmacological options for PTSD, both with limited efficacy [24]. Research on using psychedelics to treat PTSD has skyrocketed and looks promising, though more research is needed to determine efficacy and validate safe implementation procedures [25, 26]. Psychotherapeutic treatments have shown a greater benefit than pharmacological intervention alone [27]. Determining the superiority of combining psychotherapy with pharmacological treatment needs further exploration [28]. To improve the efficacy of current treatment options and to design novel, more efficacious treatment options, we must understand the neural alterations that contribute to the development of PTSD, improve with treatment, and potentially define biotypes of the disorder.

## Current narrative review

**Fig. 1:**
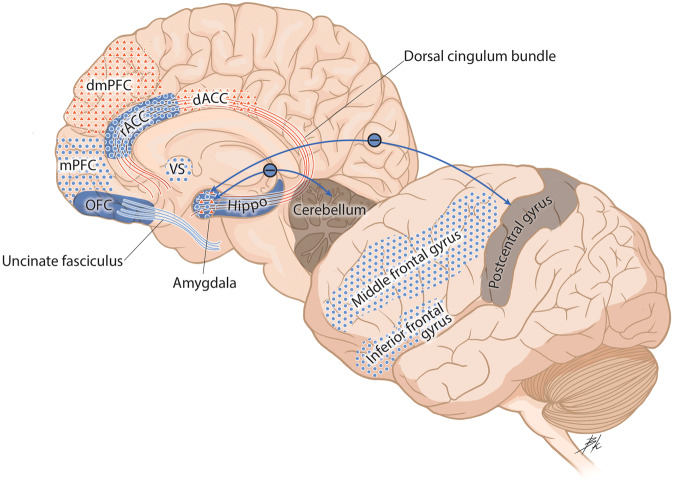
A pictorial overview of findings from Question 1: which neural alterations serve as predisposing (pre-exposure) risk factors for PTSD development, and which are acquired (post-exposure) alterations? Blue dots represent decreased activation. Red triangles represent increased activation. Solid blue represents decreased volume. Solid blue lines represent decreased structural integrity. Red lines represented increased structural integrity. Functional connectivity findings are depicted with arrows, with blue lines (–) that represent decreased functional connectivity and red lines (+) that represent increased functional connectivity. dACC dorsal anterior cingulate cortex, dmPFC dorsomedial prefrontal cortex, Hippo hippocampus, mPFC medial prefrontal cortex, OFC orbitofrontal cortex, rACC rostral anterior cingulate cortex, VS ventral striatum.

**Fig. 2:**
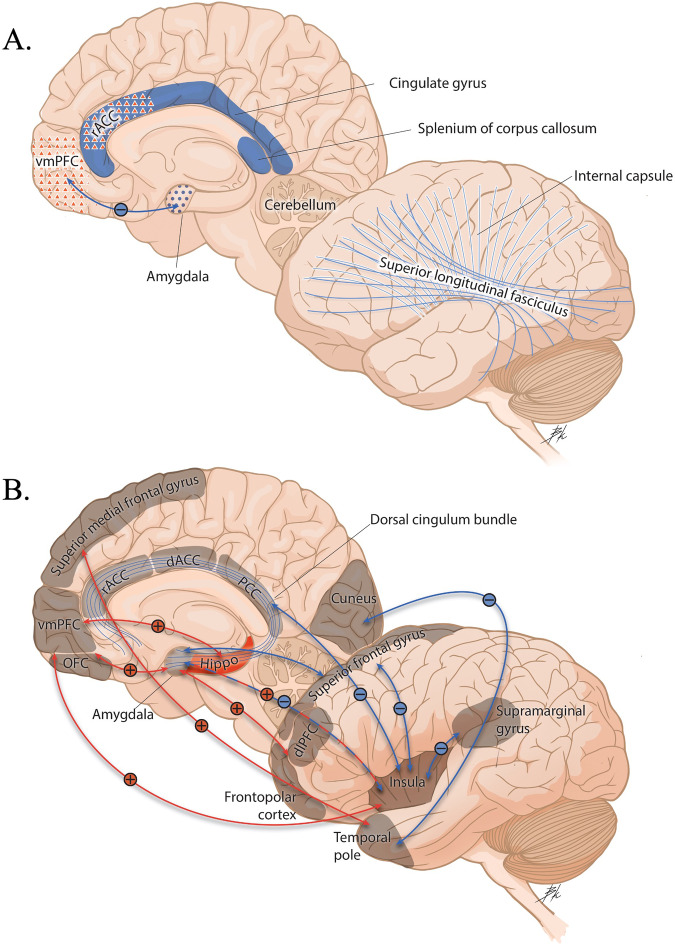
A pictorial overview of findings from Question 2: Which neural alterations can predict treatment outcomes and define clinical improvement? **A** Brain measures at baseline that predict a positive treatment response. **B** Brain measures associated with a positive response to treatment. Blue dots represent decreased activation. Red triangles represent increased activation. Solid blue represents decreased volume. Solid red represents greater volume. Solid blue lines represent decreased structural integrity. Functional connectivity findings are depicted with arrows, with blue lines (–) equating to decreased functional connectivity and red lines (+) equating to greater functional connectivity. dACC dorsal anterior cingulate cortex, dlPFC dorsolateral prefrontal cortex, Hippo hippocampus, OFC orbitofrontal cortex, PCC posterior cingulate cortex, rACC rostral anterior cingulate cortex, vmPFC ventromedial prefrontal cortex.

Given the influx of neuroimaging data published since the first neuroimaging paper using a PTSD sample in 1995 [29], an extensive library of reviews and meta-analyses has examined this literature [30–40]. Our narrative review builds upon this previous literature by exploring progress made in the past decade on three major questions in the field: (1) Which neural alterations serve as predisposing (pre-exposure) risk factors for PTSD development, and which are acquired (post-exposure)? (2) Which neural alterations can predict treatment outcomes and define clinical improvement? and (3) Can neuroimaging measures be used to define brain-based biotypes of PTSD? This review will examine structural and functional magnetic resonance imaging (MRI) literature using univariate, bivariate, and network-based approaches in adult and youth PTSD populations. Following a brief overview of neural alterations in PTSD, we will address each question above by synthesizing current findings, identifying gaps, and discussing limitations. We included articles published in peer-reviewed journals and were found using in-house expertise and searches of databases including PubMed. We will conclude by highlighting the review’s limitations and suggest future directions. Tables 1–2 provide an overview of neuroimaging findings for questions 1 and 2 (respectively), and Figs. 1–2 illustrate these findings. We will begin by describing different neuroimaging techniques.

**Table 1:** Studies that assess neural alterations that are predisposing (pre-exposure) risk factors for PTSD development or acquired (post-exposure).

Study ID	Task	Patient sample (n)	Comparison sample (n)	Trauma type	Region of interest	Direction of finding
Longitudinal Prospective Studies						
Structural Studies						
Adults						
Admon et al. [68]	sMRI	Soldiers whose hippocampus volume decreased after trauma exposure (*n* = 22; M)	Soldiers whose hippocampus volume increased after trauma exposure (*n* = 11; M)	Combat-related	Hippocampus	↓
Koch et al. [116]	sMRI	Baseline (*n* = 321; 161 M/160 F)	Follow-up (*n* = 204; 152 M/52 F)	Police-related	Hippocampus (dentate gyrus)	↓
—	—	—	—	—	Basal nucleus of amygdala	↑
Sekiguchi et al. [115]	sMRI	(*n* = 42; 33 M/9 F)	—	Natural disaster (earthquake)	vACC	↓
—	—	—	—	—	OFC	↓
Sekiguchi et al. [114]	sMRI	(*n* = 37; 28 M/9 F)	—	Natural disaster (earthquake)	Hippocampus	↓
Functional Studies						
Adults						
Admon et al. [68]	FC during backward masked photographs of military medical or civilian content	Soldiers whose hippocampus volume decreased after trauma exposure (*n* = 22; M)	Soldiers whose hippocampus volume increased after trauma exposure (*n* = 11; M)	Combat-related	Hippocampus-vmPFC	↓
Admon et al. [132]	Risky anticipation of punishment	(*n* = 24; 12 M/12 F)	—	Combat-related	Amygdala	↑
—	Reward outcome	—	—	—	Nucleus accumbens	↓
Zhang et al. [133]	rs-FC	(*n* = 321; 142 M/48 F)	—	Police-related	Salience network-anterior cerebellum	↑
Longitudinal Post-trauma Studies						
Structural Studies						
Adult						
Ben-Zion et al. [119]	sMRI	(*n* = 171; 84 M/87 F)	—	MVA (*n* = 108); Bicycle accidents (*n* = 13); Physical assaults (*n* = 11)	Hippocampus	↓
Ben-Zion et al. [120]	sMRI	Remission (*n* = 71; 33 M/38 F)	Non-remission (*n* = 29; 11 M/18 F)	MVA (*n* = 89); Assault/brawl (*n* = 5); Other (*n* = 6)	Subiculum	↓
—	—	—	—	—	CA1	↓
Fani et al. [124]	sMRI	No PTA at 6 months (*n* = 21; 12 M/9 F)	PTSD (*n* = 10; 5 M/5 F)	MVA (*n* = 24); Pedestrian accident (*n* = 3); Bicycle accident (*n* = 2); Sexual assault (*n* = 1)	Uncinate fasciculus	↓
Lindgren et al. [121]	sMRI	Low perceived stress (*n* = 76; 45 M/31 F)	Moderate to high perceived stress (*n* = 35; 18 M/17 F)	—	Hippocampus	↓
Harnett et al. [123]	sMRI	(*n* = 109; 33 M/76 F)	—	MVA (*n* = 85); Non-motorized collision (*n* = 1); Fall<10 feet (*n* = 4); Burn (*n* = 1); Animal-related (*n *= 1)	Uncinate Fasciculus	↓
Kennis et al. [125]	sMRI	(*n* = 57; M)	—	Combat-related	Dorsal cingulum	↑
Weis, Webb et al. [122]	sMRI	(*n* = 215; 118 M/97 F)	—	Mixed	Hippocampus	No change
Xie et al. [117]	sMRI	(*n* = 44; 13 M/31 F)	—	MVA	Hippocampus	↓
Quidé et al. [118]	sMRI	(*n* = 27; F)	—	Sexual assault	Hippocampus	↓
Functional Studies						
Adult						
Belleau et al. [139]	rs-FC	TE (*n* = 54; 19 M/35 F)	—	MVA (*n* = 41); Physical Assault (*n* = 10); Other type of non-vehicular incident (*n* = 3)	Amygdala-cerebellum	↓
—	—	—	—	—	Amygdala-post central gyrus FC	↓
Ben-Zion et al. [140]	Gambling task Reward > punishment	Timepoint 1 (*n* = 132; 69 M/63 F) Timepoint 2 (*n* = 115; 60 M/55 F)	Timepoint 3 (*n* = 112; 56 M/56 F)	MVA (*n* = 118); Assault/brawl (*n* = 10); Other trauma types (*n* = 5)	Ventral striatum	↓
—	Reward > punishment	—	—	—	Amygdala	↓
Du et al. [143]	rs-FC	Baseline (*n* = 21; 13 M/8 F); Follow-up (*n* = 21; 13 M/8 F)	HC (*n* = 21; 13 M/8 F)	Natural disaster (earthquake)	Fronto-limbic-striatal network-DMN	↑
Harnett et al. [141]	Pavlovian fear conditioning task	TE (*n* = 20; 14 M/6 F)	HC (*n* = 19; 14 M/5 F)	MVA (*n* = 9); Fall (*n* = 5); Burn (*n* = 3); Knife stab wound (*n* = 1); Animal accident (*n* = 1); Mechanical accident (*n* = 1)	PFC	↓
—	—	—	—	—	Inferior parietal lobe	↓
Kennis et al. [125]	Emotional processing task -Negative images	Short-term follow-up (*n* = 28; M)	Long-term follow up (n = 28; M)	Combat-related trauma	dACC	↑
Powers et al. [142]	Stop-signal anticipation task -Reactive inhibition	TE (n = 23; 15 M/8 F)	—	MVA (*n* = 12); pedestrian versus auto (*n* = 5); Assault (*n* = 1); Gunshot wound (*n* = 1); Stabbing (*n* = 1); Industrial/home accident (*n* = 1); Animal bite/attack (*n* = 1); Bike accident (*n* = 1)	rIFG	↓
—	Response inhibition	—	—	—	vmPFC	↓
Stevens et al. [138]	Fearful face processing task - Fearful > neutral	TE (*n* = 31; 16 M/15 F)	—	MVA (*n* = 22); Pedestrians hit by vehicle (*n* = 1); Motorcycle or bicycle accident (*n* = 3); Sexual assault (*n* = 2)	Amygdala	↑
—	Fearful > neutral	—	—	—	vACC	↓
Tanriverdi et al. [135]	Fearful face processing task – Fearful > neutral	TE (*n* = 116; 40 M/76 F)	—	MVA (*n* = 87); Physical assault (*n* = 15); Sexual assault (*n* = 2); Fall (*n* = 6); Nonmotorized collision (*n* = 2); Burns (*n* = 1); Other (*n* = 4)	Hippocampus	↓
van Rooij et al. [136]	Go/NoGo task -Nogo>go	Original sample (*n* = 27; 14 M/13 F)	Replication sample (*n* = 31; 20 M/11 F)	MVA (*n* = 38); Motorcycle collision (*n* = 1); Non-sexual assault (*n* = 2); Pedestrian vs. auto (*n* = 7); Industrial/home accident (*n* = 3); Bicycle accident (*n* = 3); Sexual assault (*n* = 3)	Hippocampus	↓
van Rooij et al. [134]	Fear inhibition task	TE (*N* = 28; 18 M/10 F)	—	MVA (*n* = 13); Bike accidents (*n* = 1); Non-sexual assault (*n* = 1); Sexual assault (*n* = 1); Pedestrian versus auto (*n* = 7); Gunshot wound (*n* = 1); Stabbing (*n* = 1); Industrial/home accident (*n* = 2); Animal bite/attack (*n* = 1)	Hippocampus	↓
Wang et al. [137]	Fearful face processing	PTSD (*n* = 16; 6 M/10 F)	TENC (*n* = 22; 6 M/16 F)	MVA (*n* = 38)	dmPFC	↑
Youth						
George et al. [148]	Emotion processing task - Threatening images	PTSD (*n* = 23; 13 M/10 F)	Typically developing youth (*n* = 28; 9 M/19 F)	Sexual abuse (*n* = 11); Accident (*n* = 3); Traumatic news (*n* = 4); Witness domestic violence (*n* = 5)	Hippocampus	↑
—	Neutral images	—	—	—	Hippocampus	↓
Three Group Studies						
Structural Studies						
Adults						
Luo et al. [127]	sMRI	PTSD (*n* = 57; 20 M/37 F)	TENC (*n* = 11; 6 M/5 F); HC (*n* = 39; 19 M/20 F)	Lost only child	Hippocampus	↓
Luo et al. [126]	sMRI	PTSD (*n* = 57; 20 M/37 F)	TENC (*n* = 11; 6 M/5 F); HC (*n* = 39; 19 M/20 F)	Lost only child	CA2	↓
—	—	—	—	—	CA3	↓
—	—	—	—	—	CA4	↓
—	—	—	—	—	Dentate gyrus	↓
—	—	—	—	—	Subiculum	↓
Postel et al. [130]	sMRI	PTSD (*n* = 53; 22 M/31 F)	TENC (*n* = 39; 21 M/18 F); HC (*n* = 56; 26 M/30 F)	Terrorist attack	Hippocampus	↓
van Rooij et al. [129]	sMRI	PTSD (*n* = 47; M)	TENC (*n* = 25; M) HC (*n* = 25; M)	Combat-related	Hippocampus	↓
Zhang et al. [128]	sMRI	PTSD (*n* = 69; 22 M/47 F)	TENC (*n* = 76; 20 M/56 F); HC (*n* = 57; 23 M/33 F)	Natural disaster (earthquake)	Hippocampus	↓
—	—	—	—	—	Amygdala	↓
Functional Studies						
Adult						
Chen et al. [145]	rs-FC	PTSD (*n* = 27; 7 M/20 F)	TENC (*n* = 33; 7 M/26 F); HC (*n* = 30; 7 M/23 F)	Natural disaster (typhoon)	dACC-postcentral gyrus	↑
Sullivan et al. [144]	Think-no-think task	PTSD (*n* = 16; 15 M/1 F)	TENC (*n* = 19; 18 M/1 F); HC (*n* = 13; 11 M/2 F)	Combat-related trauma (*n* = 10); Adult physical/sexual assault (*n* = 1); Accident/MVA/fire (*n* = 4); Death of someone (*n* = 1)	Middle Frontal Gyrus	↓
Twin Studies						
Adult						
Dahlgren et al. [146]	Stressful > neutral script-driven imagery	ExP+ (*n* = 12; M) UxP+ (n = 12; M)	ExP- (*n* = 14 M) UxP- (*n* = 14 M)	Combat-related	MFG	↓
Hinojosa et al. [147]	Emotional face viewing	ExP+ (*n* = 12; M) UxP+ (n = 12; M)	ExP- (*n* = 15 M) UxP- (*n* = 15 M)	Combat-related	Amygdala	↑
—	—	—	—	—	MFG	↓

*CA* Cornu ammonis, *dACC* dorsal anterior cingulate cortex, *DMN* default mode network, *dmPFC* dorsomedial prefrontal cortex, *ExP* *+*  trauma-exposed PTSD, *ExP-* trauma-exposed non-PTSD, *F* female, *FC* functional connectivity, *HC* healthy control, *M* male, *MFG* medial frontal gyrus, *MVA* motor vehicle accident, *OFC* orbitofrontal cortex, *PFC* prefrontal cortex, *PTA* post-trauma anhedonia, *PTSD* posttraumatic stress disorder, *rIFG* right inferior frontal gyrus, *rs-FC* resting-state functional connectivity, *sMRI* structural magnetic resonance imaging, *TE* trauma-exposed, *TENC* trauma-exposed non-PTSD control, *UxP* *+*  trauma-unexposed PTSD cotwin, *UxP-* trauma-unexposed non-PTSD cotwin, *vACC* ventral (rostral) anterior cingulate cortex, *vmPFC* ventromedial prefrontal cortex.

**Table 2:** Neural predictors of positive treatment response and change after treatment.

Study ID	Task	Patient Sample	Control Sample	Trauma Type	Region of Interest	Direction of Finding	Treatment type
Baseline Predictors of PTSD Symptom Improvement							
Structural Studies							
Adult							
Graziano et al. [152]	DTI-FA	PTSD (*n* = 21; F)		Interpersonal violence	Internal capsule	Lower FA at baseline predicted greater symptom reduction	12 weeks of CPT; treatment improvement was used using change scores: posttreatment-pretreatment CAPS
—	—	—	—	—	Posterior limb of the internal capsule	Lower FA at baseline predicted greater symptom reduction	—
—	—	—	—	—	Cingulate gyrus	Lower FA at baseline predicted greater symptom reduction	—
—	—	—	—	—	Superior longitudinal fasciculus	Lower FA at baseline predicted greater symptom reduction	—
—	—	—	—	—	Splenium of the corpus callosum	Lower FA at baseline predicted greater symptom reduction	—
Functional Studies							
Adult							
Hinojosa et al. [153]	Emotional face-viewing	PTSD (*n* = 16; 6 M/10 F)	—	—	Amygdala	Lesser pre-treatment amygdala activation in response to fearful vs. happy facial expressions was each related to greater symptomatic improvement with PE A greater decline in amygdala responses from the first to the last fearful facial expression block was associated with greater improvement	8 sessions of PE; treatment improvement was measured using change scores; posttreatment-pretreatment SPRINT.
—	—	—	—	—	rACC/vmPFC	Greater pre-treatment activation in response to fearful versus happy facial expressions was each related to greater symptomatic improvement with PE	—
Korgaonkar et al. [157]	Pairwise intrinsic task-free FC	PTSD (*n* = 36; 19 M/17 F) Treatment responders (*n* = 25; 12 M/13 F) Treatment non-responders (*n* = 11; 7 M/4 F)	HC (*n* = 36; 18 M/18 F)	Childhood abuse (*n* = 3); MVA (*n* = 5); police-related (*n* = 10); assault (*n* = 14); witness (*n* = 3)	Cingulo-opercular, DMN, dorsal attention, and salience networks	Lower pre-treatment intranetwork intrinsic connectivity is associated with treatment improvement	9 sessions of TF-CBT; Responders defined as having at least 50% improvement in symptoms.
—	—	—	—	—	Cingulo-opercular, DMN, dorsal attention, and frontoparietal networks	Lower pre-treatment intranetwork intrinsic connectivity is associated with treatment improvement	—
—	—	—	—	—	Cingulo-opercular, dorsal attention, and frontoparietal networks with auditory and visual networks	Lower pre-treatment intranetwork intrinsic connectivity is associated with treatment improvement	—
—	—	—	—	—	Basal ganglia regions of the subcortical network with the DMN, cingulo-opercular, frontoparietal, and salience networks	Lower pre-treatment intranetwork intrinsic connectivity is associated with treatment improvement	—
—	—	—	—	—	Visual and somatomotor networks	Lower pre-treatment intranetwork intrinsic connectivity is associated with treatment improvement	—
Norbury et al. [154]	Emotional face-viewing	PTSD Midazolam (*n* = 10; 2 M/8 F)	PTSD Ketamine (n = 10; 1 M/10 F)	Sexual violence (*n* = 10); physical violence or abuse (*n* = 6); witnessed violence or death; combat exposure (*n* = 4);	vmPFC-amygdala connectivity	Lower baseline was related to greater PTSD symptom improvement	Midazolam versus Ketamine (Drug treatment); change scores were positive meaning baseline minus outcome visit scores.
—	—	—	—	—	rACC	Lower baseline during emotional face-viewing and emotional conflict regulation tasks and in individuals with more distinct representation of fearful vs. neutral faces across rACC voxels	—
Yuan et al. [156]	rs-fMRI	PTSD (*n* = 22; 5 M/17 F) Remitted (*n* = 9; 1 M/8 F) Persistent patients (*n* = 11; 4M7F)	—	Natural disaster (earthquake)	Precuneus	Discriminating region in remitted versus persistent patients revealed by combined ALFF and DC	12 weeks of treatment with paroxetine; remitted patients defined by a CAPS improvement of 50% or greater, persistent patients with <50% improvement.
—	—	—	—	—	dmPFC	Discriminating region in remitted versus persistent patients revealed by combined ALFF and DC	—
—	—	—	—	—	Frontal orbital cortex	Discriminating region in remitted versus persistent patients revealed by combined ALFF and DC	—
—	—	—	—	—	Supplementary motor area	Discriminating region in remitted versus persistent patients revealed by combined ALFF and DC	—
—	—	—	—	—	Cerebellum	Discriminating region in remitted versus persistent patients revealed by combined ALFF and DC	—
—	—	—	—	—	Lingual gyrus	Discriminating region in remitted versus persistent patients revealed by combined ALFF and DC	—
Youth							
Cisler et al. [161]	Cognitive reappraisal task – Reappraisal during negative images > viewing negative images	PTSD (*n* = 34; F)		Assaultive violence	Amygdala-insula FC	Decreased FC	12 sessions of TF-CBT.
Garrett et al. [158]	Facial expression task with scrambled images	PTSD (*n* = 20; 2 M/18 F)	HC (n = 20; 2 M/18 F)	Interpersonal violence	Posterior cingulate	Lower activation predicted better treatment	12 sessions of TF-CBT. Symptom improvement based on 50% or better.
—	—	—	—	—	Mid-cingulate	Lower activation predicted better treatment	—
—	—	—	—	—	Hippocampus	Lower activation predicted better treatment	—
Zhutovsky et al. [159]	rs-FC	Responders (*n* = 21; 10 M/12 F)	Non-responder (n = 19; 5 M/14 F)	Sexual abuse (*n* = 13); Domestic violence (*n* = 5); Community violence (*n* = 10); Accidents/Medical (*n* = 5); Other (*n* = 7)	Superior temporal gyrus	Network distinguished between responders and non-responders with 76.2% accuracy	TF-CBT or EMDR. Improvement rated 30% symptoms
Brain Changes Related to Symptom Improvement							
Structural Studies							
Adult							
Bossini et al. [163]	VBM	PTSD (*n* = 19; 9 F/10 M)		Natural disaster (*n* = 3); sudden death of a family member (*n* = 5); MVA (*n* = 2); assault/robbery (*n* = 6); terrorist attack (*n* = 4)	Parahippocampal gyrus	Increase in volume	12 EMDR sessions over three months
—	—	—	—	—	Thalamus	Decrease in volume	—
Butler et al. [165]	VBM	PTSD (*n* = 20; M)	—	Combat-related	Hippocampus	Increase in volume	12 EMDR sessions; patients assigned to a therapy group or a wait-list control group
Butler et al. [164]	VBM	PTSD (*n* = 40; M) EMDR+Tetris (*n* = 20; M) EMDR (*n* = 20; M)	—	Combat-related	Hippocampus	Whole-brain analysis: Significant increase in GMV after therapy in the Tetris group. ROI analysis: Compared to the control group, larger volumes in the hippocampus were found in the Tetris group after therapy	Every day for 6 weeks Tetris + 12 EMDR sessions versus 12 EMDR session only group
Kennis et al. [166]	DTI	PTSD (*n* = 39; M)	TENC (*n* = 22; M)	Combat-related	Dorsal cingulum	After treatment, higher FA values in the dorsal cingulum were found in patients with persistent PTSD versus patients with remitted PTSD and combat controls	Approx. 9 sessions Trauma-focused therapy, TF-CBT or EMDR
Levy-Gigi et al. [162]	Volumetric analyses	PTSD (*n* = 39; 30 F/9 M)	TENC (*n* = 31; 20 F/11 M)	Environmental disaster (*n* = 22); violent crime (*n* = 13); traffic accident (*n* = 23); combat (*n* = 6); emergency service workers (*n* = 6)	Hippocampus	Clinical improvement during CBT in PTSD was associated with increased hippocampal size and elevated FKBP5 gene expression. And these values were significantly correlated with clinical improvement (though FKBP5 was the primary predictor)	12 weekly 1.5 hour Trauma-focused CBT sessions
Functional Studies							
Adult							
Fonzo et al. [168]	DCM-effective connectivity	PTSD (*n* = 66) Immediate Treatment (n = 36; 13/23 F)	Patient waitlist (*n* = 30; 10 M/20 F)	Natural disaster (*n* = 4); physical assault (*n* = 16); assault w/ weapon (*n* = 5); sexual assault (*n* = 21); combat exposure (*n* = 8); injury/illness/suffering (*n* = 12)	Amygdala	Treatment decreased left frontal inhibition of the amygdala and larger decreases were associated with larger symptom reductions	9 to 12 sessions of PE or Treatment waiting list
Leroy et al. [169]	Granger causality	PTSD Responders (*n* = 16; 7 M/9 F)	PTSD Non-responders (**n** = 14; 8 M/6 F)	Life threat (*n* = 2); MVA, work accident (*n* = 3); physical assault (*n* = 3); sexual trauma/rape (*n* = 5); Terrorist attack (*n* = 3)	Anterior insula - superior frontal gyrus, anterior and posterior supramarginal gyri, anterior and posterior cingulate, central operculum and right amygdala	Reduced influence showed greater clinical improvement	Traumatic memory reactivation therapy + propranolol or traumatic reactivation therapy + placebo. Once a week for 6 consecutive weeks; having at least a 33% decrease in PCL-5 questions 1-to-5 score compared to baseline
Korgaonkar et al. [157]	Pairwise intrinsic task-free FC	PTSD (*n* = 36; 19 M/17 F) Treatment responders (n = 25; 12 M/13 F) Treatment non-responders (n = 11; 7 M/4 F)	HC (n = 36; 18 M/18 F)	Childhood abuse (*n* = 3); MVA (*n* = 5); police-related (*n* = 10); assault (*n* = 14); witness (*n* = 3)	Somatomotor and visual networks	Connectivity increased in treatment responders from pre-to post-treatment	9 sessions of TF-CBT; Responders defined as having at least 50% improvement in symptoms
Santarnecchi et al. [171]	rs-FC	PTSD (*n* = 37; 19 M/12 F) TF-CBT (*n* = 14; 9 M/5 F) EMDR (*n* = 17; 10 M/7 F)	—	Natural disaster (earthquake)	Superior medial frontal gyrus – temporal pole	Increased connectivity	TF-CBT (10 sessions) and EMDR (4 sessions);
—	—	—	—	—	Cuneus-temporal pole	Decreased connectivity	—
Vuper et al. [167]	rs-FC	PTSD Treatment completers (*n* = 26; F)	PTSD Intent-to-treat (n = 42; F) TENC (n = 18; F)	Interpersonal violence	DMN	Decreased connectivity in PTSD participants after CPT	12 sessions of CPT; intent-to-treat sample
—	—	—	—	—	CEN	Normalization of CEN connectivity with treatment	
Zhu et al. [170]	rs-FC	PTSD (*n* = 24; 7 M/17 F)	TENC (*n* = 26; 7 M/19 F)	MVA; sexual or physical assaults; witnessing serious injuries or deaths	BLA, CMA - OFC	Increased rs-FC among patients with PTSD, but not among TENC	10 sessions of PE
—	—	—	—	—	Hippocampus-mPFC	Increased rs-FC among patients with PTSD, but not among TENC	

*ALFF* amplitude of low-frequency fluctuations, *BLA* basolateral amygdala, *CAPS* clinician-administered PTSD scale, *CEN* central executive network, *CMA* centromedial amygdala, *CBT* cognitive behavioral therapy, *CPT* cognitive processing therapy, *DC* degree centrality, *DCM* dynamic causal modeling, *DMN* default mode network, *dmPFC* dorsomedial prefrontal cortex, *DTI* diffusion tensor imaging, *EMDR* eye-movement desensitization reprocessing therapy, *F* female, *FA* fractional anisotropy, *FC* functional connectivity, *HC* healthy control, *M* male, *MVA* motor vehicle accident, *mPFC* medial prefrontal cortex, *OFC* orbitofrontal cortex, *PE* prolonged exposure therapy, *PTSD* posttraumatic stress disorder, *rACC* rostral anterior cingulate cortex, *rs-FC* resting-state functional connectivity, *SPRINT* short PTSD rating interview, *TENC* trauma-exposed non-PTSD control, *TF-CBT* trauma-focused cognitive behavioral therapy, *VBM* voxel-based morphometry, *vmPFC* ventromedial prefrontal cortex.

## Neuroimaging techniques

### Structural techniques

Structural MRI of PTSD populations typically determines alterations in the morphometry of brain regions in patients versus controls (trauma-exposed non-PTSD [TENC] or healthy controls [HC]). Morphometric measures include subcortical and cortical gray matter volumes, thickness, and white matter microstructure. There are two analysis pipelines one can follow: (1) surface-based, which identifies borders between pial and white matter surfaces, and (2) voxel-based, which labels each voxel in cortical and subcortical tissues and allows for calculating subcortical structures and total intracranial volume [41]. Thus, structural volume can be measured by contrasting volume inside the pial surface from the white surface and regions, not part of the cortex, or by measuring total cortical labeled voxels. Cortical thickness is measured by contrasting the distance between the pial and the white surface [41]. Generally, smaller volumes and lower cortical thickness are representative of poorer structural integrity. Diffusion tensor imaging is a structural tool used to measure the structural integrity of white matter tracts via the diffusivity of water molecules along the axial direction of white matter fibers. The pattern of diffusivity can be computed using scalar measures such as fractional anisotropy (FA), whereby lower FA values illustrate a reduced axonal packing density [42].

### Functional techniques

Functional MRI (fMRI) can be used as an indirect proxy to measure brain activation with great spatial and limited temporal resolution. Researchers record activation during the presentation of different tasks designed to induce activation in regions responsible for given functions. There are many different approaches one can take to analyze fMRI data, which can be categorized into three general techniques. The simplest technique is univariate analyses, which examine the activation of single voxels in response to various tasks. As techniques developed with time, more researchers have also included bivariate analyses, which calculate the temporal association of two regions based on activation. This includes task-based and resting-state functional connectivity (FC), which measure spontaneous changes in brain activation during the completion or absence of a task, respectively. Finally, recent years have seen an increase in network-based approaches, which measure activation across many brain regions and networks. Network-based approaches in fMRI studies typically conceptualize the human brain as a network of interconnected functional components that operate in a coordinated dynamic fashion [43]. By employing such methods, researchers can overcome the limitations of traditional univariate and bivariate approaches by mitigating the bias of preselecting target regions a priori and allowing a more comprehensive investigation of large-scale brain organization (rather than isolated regions or simplistic circuits). In this narrative review, we will focus on the two primary tools for network-based analysis of neuroimaging data in PTSD: (1) independent component analysis (ICA), which isolates individual functional networks within the whole brain, and (2) graph theory methods, which examine properties of networks (e.g., nodes and edges of a graph) and characterize them based on their intercorrelations.

## Neural alterations in PTSD

### Adults

PTSD is the only DSM diagnosis with a known origin (i.e., experiencing trauma). Given the importance of traumatic memory in the development of the disorder, early neuroimaging studies sought to discover alterations in structure and function of brain regions implicated in fear learning and memory (e.g., amygdala and hippocampus) in patients with PTSD compared to controls. These discoveries have created “classical” neurocircuitry models of PTSD that emphasize an inability of cortical regions to successfully regulate subcortical regions important in initiating a fear response [33]. Research over the last decade has noted diminished structural integrity in areas associated with executive functions, including reduced cortical volumes in the anterior cingulate cortex (ACC) [44, 45] and frontal cortical regions [44, 46] in PTSD patients and in subcortical structures, including the amygdala and hippocampus (see [39, 47] for recent reviews). Furthermore, PTSD patients, compared to controls, have shown reduced white matter integrity in the uncinate fasciculus (UF) [48–50], corpus callosum, corticospinal tract, and enhanced white matter integrity in the inferior fronto-occipital fasciculus, and inferior temporal gyrus (for review see [51]), highlighting the structural disconnect between cortical and subcortical regions. Functional studies also support classical neurocircuitry models, with greater amygdala alongside reduced activation in brain regions associated with emotional regulation (e.g., ventromedial prefrontal cortex [vmPFC], inferior, superior, medial frontal gyrus [MFG], ACC, dorsolateral PFC [dlPFC], and dorsomedial PFC [dmPFC]) during emotion-related tasks [52–62], extinction recall [63], and fear generalization [64, 65]. Limitations of these classical neurocircuitry models of PTSD include the deficiency of the models to understand the biological bases of PTSD systems holistically rather than focusing on fear processes alone (see [35] for an in-depth discussion).

Network-level neural alterations in PTSD are present. Specifically, disruptions in connectivity structure or activation profiles within the salience network (SN), default mode network (DMN), and central executive network (CEN) may underlie univariate and bivariate impairments in PTSD patients. For example, hyperarousal and hyperreactivity symptoms were linked to increased activation of the amygdala and dACC, two critical nodes of the SN [33, 66, 67]. Further, intrusive symptoms, impaired fear extinction, and deficits in emotional regulation are associated with decreased activation of the hippocampus and vmPFC, two nodes of the DMN [31, 33, 68]. Decreased activation of nodes within the CEN, such as the IFG and MFG in PTSD, are present [32].

Numerous neuroimaging studies have employed ICA methodology to test alterations in large-scale networks in adults with PTSD (see reviews [69–74]). Overall, results are mixed and provide limited support for classical neurocircuitry models of PTSD. Within the DMN, most studies report decreased activation and connectivity at rest in PTSD patients [75–77], possibly associated with re-experiencing and dissociative symptoms [71, 78]. However, one study reported higher integration of the amygdala with the DMN in PTSD patients during a threat-processing task [79]. Research typically suggests increased activation and connectivity [83, 84] within the SN, potentially linked to hyperarousal and hypervigilance symptoms. In contrast, other findings indicate decreased SN connectivity in PTSD [48, 77]. The CEN seems to show reduced activation and connectivity among PTSD patients [32, 76].

In addition to alterations within each network, some recent evidence points to aberrant connectivity patterns between networks in PTSD. For example, Zhang and colleagues (2015) [77] reported decreased FC between the SN and DMN, possibly explaining previous contrasting findings regarding the connectivity of the SN in PTSD. As the SN is believed to facilitate the transition between the DMN and CEN in response to external cognitive demands [80], the reduced connectivity between the SN and DMN might suggest a compromised ability in PTSD to shift between a self-referential state and a cognitive control mode. In another study, PTSD patients showed increased excitatory influence of the executive central network (ECN, like the CEN) on the posterior DMN. Finally, Akiki et al. (2017) [71] suggested that PTSD is characterized by impaired SN, incapable of DMN-CEN modulation, and weakened top-down regulation of the SN by the CEN.

Several neuroimaging studies of PTSD used graph theory approaches in resting-state data to examine possible alternations in local and global connectivity patterns. One study found that PTSD patients exhibit a transformation from a random or regular network to a “small-world” network, compared to TENC [81]. The concept of “small-world” networks describes a network topology in which most nodes are not neighbors of one another, but still, nodes can be reached from every other by a small number of steps [82]. Furthermore, these patients show increased centrality in the DMN and SN [81] (i.e., amount of nodes with many paths passing through them). Indeed, more severe PTSD symptoms were linked to DMN alteration, including decreased FC strength [83], decreased functional integration, and increased segregation within the DMN [84]. Additionally, reduced within-network connectivity and decreased connectional density within a hippocampus-PFC network are associated with more severe re-experiencing symptoms in combat-exposed veterans [85]. These studies suggest a complex interplay of network alterations in PTSD.

### Youth

In normative brain development, gray matter volume is shaped like an inverted U, whereby matter increases from birth to childhood, then around early adolescence, starts to decline until adulthood [86]. This development pattern is due to synaptic proliferation and pruning, which makes the child’s brain more efficient [87]. White matter generally increases throughout childhood and adolescence and then levels off [86]. This pattern represents increases in myelination over time to maximize neuronal transmission speed and adjust the timing and synchrony of neural spikes [88]. In total volume, including gray and white matter, young brains grow in size until late childhood/early adolescence, when they start to asymptote [89].

In pediatric samples, PTSD and TENC youth typically exhibit overall smaller amygdala [90–92] and hippocampal volumes [91, 93], including smaller CA2/3 hippocampal subfields [90, 94]. Studies report that youth with PTSD have smaller vmPFC volumes than TENC or HC youth [91, 95]. One study found no differences in the structure of the medial PFC (mPFC) between youth with PTSD and TENC [96], indicating that more work needs to be done to disentangle how maltreatment and PTSD relate to volume. Heyn and colleagues [97] (2022) explored sex differences in volume in female and male youth. Female youth with PTSD showed increased volume and surface area in the ventrolateral PFC and frontal pole regions. In contrast, male youth showed smaller volumes of these regions that predicted more severe symptoms one year later [97]. Additionally, youth with PTSD, compared to controls, had age- and sex-related differences in the UF, inferior longitudinal fasciculus, and cingulum bundle [98]. Finally, a review found that youth with PTSD had lower FA in the corpus callosum, including the anterior and posterior midbody, the isthmus, and the splenium [99], and increased PTSD symptoms have been related to lower FA in these regions [100].

Our comprehension of the typical developmental trajectories of brain function in youth remains limited. Univariate investigations have focused mostly on associations between childhood trauma and brain activation. In the past decade, a movement towards employing consistent brain atlases and pre-processing methods has emerged, particularly for comparing studies with limited sample sizes [101]. That said, the literature reviewed here will be specific to youth with PTSD and is, therefore, sparse.

In task-based fMRI, greater activation in the amygdala and dACC in response to emotional faces and threatening images have been found in youth with PTSD versus controls [102, 103]. Greater activation has been found in the ACC and frontal brain regions in maltreated youth than in HC during the presentation of negative stimuli [104]. Furthermore, there is an interesting pattern of decreased FC while viewing angry faces and increased FC while viewing happy faces, specifically between the dACC-dmPFC, amygdala-dmPFC, and amygdala-vlPFC [102]. Youth with PTSD showed increased PCC-vmPFC resting-state FC, which may indicate problems in self-referential tasks or memory consolidation [105]. In another resting-state study, youth with PTSD showed decreased PCC-hippocampus FC and increased PCC-insula and PCC-cerebellum FC [106].

Network-based analyses have had limited application in youth PTSD populations. This could be for numerous reasons, including low sample sizes and the novelty of computational imaging methods. The handful of studies that have used ICA and graph theory methods to identify alterations in youth with PTSD have identified a greater anticorrelation between DMN and task-positive network (TPN), indicative of difficulty switching between internal (DMN) and external (TPN) stimuli [105]. In one study of resting-state whole-brain connectivity in youth exposed to an earthquake, the PTSD group (compared to TENC) showed an increased clustering coefficient and a normalized characteristic path length and local efficiency, suggesting a shift toward regular networks [107]. Further, the authors found enhanced nodal centralities in the DMN and SN, which may be related to altered processing of negative emotions. They also found reduced centralities in the CEN, which may indicate worse goal-directed behaviors. In contrast, Xu and colleagues (2018) [108] reported a lower clustering coefficient among youth with PTSD compared to TENC. They further found increases in centralities in the attention and DMN and decreases in the salience and sensorimotor networks [109].

## Question 1: which neural alterations serve as predisposing (pre-exposure) risk factors for PTSD development, and which are acquired (post-exposure)?

An important goal in the PTSD field is to uncover whether the neural alterations in PTSD discussed above are predisposing risk factors that make an individual more susceptible to developing PTSD after experiencing trauma or acquired characteristics of the disorder. Uncovering these distinctions will enable the development of preventative interventions or the creation of more efficacious treatment options that target specific targets affected by the disorder. Many methodological approaches are used to disentangle predisposed from acquired neural alterations in PTSD [110]. The most methodologically sound techniques include prospective longitudinal studies, which collect neuroimaging data from participants either before trauma exposure or in the early aftermath of trauma and follow these participants at various time points post-trauma. Examples include the Neurobehavioral Moderators of Posttraumatic Disease Trajectories (NMPTDT) [111] and the Advancing Understanding of Recovery After Trauma (AURORA) studies [112]. While prospective longitudinal study designs are optimal for answering this question, they are hard to execute as they are often time-consuming, expensive, and have inherently poor participant attrition rates (see [111]). Further, though it is the goal to recruit participants before or in the early aftermath of trauma, participants may endorse childhood trauma experienced years before study participation, confounding the data collected.

Twin-pair designs have also been used to answer this question. Usually, these studies include monozygotic twin pairs, where one twin has PTSD from combat-related trauma, while their co-twin did not experience combat trauma nor has a PTSD diagnosis. A separate monozygotic twin pair contains a cotwin who experienced combat-related trauma but did not develop PTSD, and their cotwin did not experience combat-related trauma nor develop PTSD [113]. Again, limitations exist in this design, including an inability to determine whether the findings are attributed to heredity or shared environments.

Lastly, while not optimal, cross-sectional studies that explore brain alterations in three groups, PTSD, TENC, and HC, can provide some insight into whether alterations are PTSD-specific or related to trauma exposure.

### Structural neuroimaging

#### Adults

Few structural imaging studies have used prospective longitudinal designs in the past decade. Studies that scanned participants pre-trauma and post-trauma found reduced hippocampal volume [68, 114], post-pre-trauma orbitofrontal cortex (OFC) volume, and pre-trauma ventral ACC were related to greater PTSD symptom severity post-trauma [115]. It is important to highlight that these studies had relatively small sample sizes (*n* < 50). One large study (*n* = 210) that scanned police recruits found smaller pre-trauma dentate gyrus volume was associated with greater PTSD symptom severity post-trauma and that experiencing more police-related trauma between scan assessments was related to an increase in the volume of the basal nucleus of the amygdala [116]. It should be noted that the studies reviewed consisted of resilient individuals, with many participants without a PTSD diagnosis.

Numerous recent longitudinal studies have investigated how structural neuroimaging data collected shortly after trauma correlate with or predict PTSD symptoms. Most of these studies supported the hypothesis that decreased hippocampal volume early post-trauma is a risk factor for the development of chronic PTSD [117–121]. However, one study found no associations between hippocampal volume, or any of its subregions, and PTSD symptoms across time [122]. These differences are likely due to differences in trauma experienced, the timing of neuroimaging measurements, analytic strategy, and other sample characteristics [122]. Reduced FA of the UF [123, 124] and greater FA of the dorsal cingulum [125] collected early post-trauma predicted greater PTSD symptoms at 3 months, 6 months, and 4 years later (respectively).

Cross-sectional studies comparing three groups - PTSD, TENC, and HC - found reduced hippocampal [126–128] and right amygdala volume [128] in the PTSD and TENC groups compared to the HC group. However, one study found that only the PTSD group showed significantly less hippocampal volume compared to the TENC and HC groups [129]. When examining hippocampal subregions, the CA1 and CA2-3/DG were significantly smaller in PTSD patients than in TENC and HC groups [130]. Differences in findings may be attributed to different trauma types endured, as has been found previously [131]. For example, the studies that found differences only in the PTSD group used samples that experienced combat-related trauma [129] or a terrorist attack [130] versus the loss of a loved one [126, 127] or a natural disaster [128].

### Functional MRI

#### Adults

Using a prospective longitudinal study design, Admon and colleagues (2013) found that service members who exhibited reduced hippocampal volume post-pre-trauma also displayed reduced hippocampus-vmPFC FC, which was related to greater PTSD symptoms post-trauma [68]. In a separate study from the same research group, the authors found that greater amygdala activation in response to risk anticipation at pre- and post-trauma was related to more PTSD symptoms post-trauma [132] and reduced nucleus accumbens activation to reward post-trauma was related to greater PTSD symptoms post-trauma [132]. Zhang and colleagues (2022) [133] recently used a network-based approach to measure stress-induced connectivity, changing patterns of large-scale brain networks at baseline to the subsequent symptom development post-trauma. In this prospective sample of police trainees, increased coupling between the SN and anterior cerebellum was observed in participants with greater PTSD symptoms (particularly intrusion symptoms) [133]. Nevertheless, as this work focused on a relatively healthy and resilient sample, future studies in more severe PTSD samples are needed.

Greater hippocampal activation, collected early post-trauma, during fear extinction [134], but not when looking at fearful versus neutral face stimuli [135], predicted more severe PTSD symptoms at 3 months post-trauma. In comparison, lesser hippocampal activation during response inhibition predicted greater PTSD symptom severity post-trauma [136]. Highlighting the unique contribution of the hippocampus in these different constructs. Greater amygdala activation early post-trauma when viewing fearful facial expressions significantly predicted symptoms at 3 [137] and 12 months post-trauma [138]. In combat veterans, greater dACC activation to negative images predicted greater PTSD symptom severity four years later [125]. Similarly, more negative amygdala-cerebellum FC at rest and amygdala-post-central gyrus FC during trauma recall at 2 weeks post-trauma predicted 6-month PTSD symptom severity post-trauma [139]. In a longitudinal study of *n* = 171 recent trauma survivors, PTSD severity at 14 months after trauma was associated with decreased neural activity in the ventral striatum (VS) and the amygdala toward rewards versus punishments at 1 month after trauma [140]. Surprisingly, decreased VS activity and connectivity with the vmPFC were more predictive of PTSD symptoms compared to the amygdala’s activity, highlighting the important role of reward processing in PTSD development or recovery [140]. Similarly, lesser activation in cortical regions early post-trauma during fear conditioning [141] and response inhibition [142] is related to greater PTSD symptoms 3 and 6 month post-trauma, respectively. However, greater dmPFC activation to fearful versus neutral face stimuli early post-trauma was associated with greater PTSD symptoms 3 months later [137]. Finally, a longitudinal study showed that while FC changes at 3 weeks post-trauma involved the DMN and frontal–limbic–striatal network, only changes in the DMN persisted at the 2 year follow-up [143].

Using a three-group design, one study examined the neural correlates of memory suppression in PTSD and found that the PTSD and TENC groups exhibited disrupted MFG activation while attempting memory suppression compared to HC, suggesting that disruptions in the MFG are apparent even in those trauma-exposed, regardless of PTSD status [144]. Regional parameters of the insular lobe, putamen, and precuneus of typhoon-related PTSD patients, TENC, were abnormal compared to HCs [145].

Recent twin studies have shown that PTSD patients exhibit reduced activation in the rostral ACC and MFG compared to their non-trauma-exposed cotwins and trauma-exposed individuals without PTSD. This diminished response, observed during exposure to trauma-related cues and to surprised faces, indicates that changes in these cortical areas are likely acquired traits of the disorder [146, 147].

#### Youth

One study found that increased hippocampal activation to threatening images over one year predicted a non-remitting PTSD trajectory, compared to a remission trajectory and HC groups [148].

### Summary

Overall, across longitudinal prospective study designs and studies utilizing three groups, hippocampal alterations (lesser hippocampal volume and function) appear to be a pre-exposure risk factor for the development of PTSD [68, 114, 116–121, 126–130, 134–136]. Creating interventions that promote hippocampal neurogenesis will be important to use to prevent the development of PTSD early after trauma. Furthermore, alterations in cortical regions such as the vmPFC, ACC, and MFG are apparent early after trauma and predictive of later PTSD symptoms [115, 125, 137, 138, 141, 142, 144–147]. As such, interventions introduced early post-trauma that promote greater FC between frontal-limbic networks can potentially strengthen these connections. There are many limitations to the studies reviewed above, including limited sample sizes, many of the longitudinal studies reviewed included participants with sub-threshold PTSD, and there was not much variability concerning trauma type. Thus, the generalizability of the findings across trauma types is questionable. For a pictorial overview of findings, see Fig. 1.

## Question 2: which neural correlates predict treatment outcomes and define treatment improvement?

To date, while trauma-focused cognitive behavioral therapies are gold-standard treatment options for PTSD, many people do not respond well to treatment [19, 20]. Uncovering the neural mechanisms that predict symptom improvement and define treatment response will be crucial in helping guide clinician-decision making and provide a more precision-medicine approach to treatment. Given the recent reviews published on this topic [40, 149–151], we review studies not included in these reviews, provide an overall summary of findings, and discuss limitations.

### Baseline prediction of PTSD symptom improvement

#### Adults

In females who developed PTSD as a result of interpersonal trauma, pre-treatment FA values of the internal capsule, cingulate gyrus, superior longitudinal fasciculus, and splenium of the corpus callosum were positively correlated with changes in PTSD symptoms after cognitive processing therapy [152]. One study showed that lesser pre-treatment amygdala activation and greater MFG to fearful versus happy facial expressions were associated with a better response to prolonged exposure therapy (PE) [153]. Furthermore, this study found a greater decrease in the amygdala activation across blocks of fearful facial expression was associated with better symptomatic improvement [153]. Lower pre-treatment vmPFC-amygdala connectivity during an emotional face-viewing task predicted symptom improvement in individuals with PTSD, an effect that was strongest in individuals who received ketamine (versus midazolam) [154]. Additionally, this study showed symptom improvement following ketamine was predicted by decreased dACC activity during an emotional conflict regulation task and an increased resting-state FC between the vmPFC and anterior insula [154]. On the other hand, ketamine did not promote a greater increase in amygdala-mPFC resting-state FC but elicited a stronger transient decrease in vmPFC-amygdala compared to midazolam [155]. A longitudinal resting-state fMRI study employing support vector machine learning highlighted the precuneus, dmPFC, lingual gyrus, supplementary motor area, and cerebellum showed the highest prognostic remittance value from paroxetine treatment [156]. Lastly, Korgaonkar and colleagues (2020) found that lower pre-treatment connectivity in the cingulo-opercular, salience, and dorsal attention networks was associated with a better response to trauma-focused cognitive behavioral therapy (TF-CBT) [157].

#### Youth

Few studies have explored whether neuroimaging measures can predict treatment response in youth diagnosed with PTSD. Decreased pre-treatment activation in the posterior cingulate, mid-cingulate, and hippocampus predicted greater symptom improvement [158]. Another study trained a support vector machine from brain networks created from an ICA, finding that the bilateral superior temporal gyrus center network distinguished between non-responders and responders to trauma-focused therapies [159]. This may indicate that auditory processing and social cognition may be important for PTSD remission [160]. Girls who experienced greater reductions in PTSD symptoms exhibited decreased amygdala-insula connectivity during reappraisal compared to those experiencing milder reductions [161].

### Neuroimaging correlates of PTSD symptom improvement

#### Adults

Increased hippocampal volume appeared in PTSD patients who completed CBT [162], eye-movement desensitization and realization (EMDR) alone [163] or paired with a Tetris video game intervention [164], and in those who remitted following psychotherapy [165]. Over time, lesser dorsal cingulum FA was found in individuals whose PTSD symptoms decreased after trauma-focused treatment [166]. Interestingly, recent work reported the normalization of CEN connectivity following cognitive processing therapy for PTSD [167]. Greater reduction in PTSD symptoms was associated with larger pre- to post-treatment increases in the inferior frontal junction inhibition of the amygdala [168]. PTSD patients who showed clinical improvement exhibited a reduced relative influence of the anterior insula over motor, affective, and self-other distinction regions [169]. Upon completion of PE, PTSD patients showed increased pre-post FC in basolateral amygdala-OFC, centromedial amygdala-OFC, and hippocampus-vmPFC. In contrast, TENC saw no significant pre-post changes in connectivity after PE, suggesting that amygdala FC normalized similarly to TENC [170]. One study showed that a reduction in PTSD symptom severity was associated with decreased connectivity between the visual cortex and temporal lobe regions and increased connectivity between the superior frontal gyrus and temporal pole regions after EMDR and TF-CBT, suggesting minor differences exist in neurophysiological outcome that is therapy-specific, particularly in those who experienced natural-disaster [171].

### Summary

Overall, the studies reviewed here and previously published reviews [40, 149–151] suggest that treatment non-response in adults was predicted by greater activation in regions responsible for threat detection, lesser activation in emotion regulation, executive function, and contextual processing regions, and altered crosstalk between regions within the DMN and regions important in emotion processing, cognitive function, and salience. In youth, studies are sparse but show a pattern of greater activation in memory-related regions, while lesser connectivity between fear learning-related regions predicted symptom reduction. There are many limitations of the studies reviewed. First, given the stringent inclusion/exclusion criteria many of these intervention studies endorse, their sample sizes are limited. Second, the definition of a responder versus a non-responder to treatment is not objective, and studies define this differently. Third, some variables are not controlled for, making it hard to determine the effect of treatment. For example, no direct comparison exists between groups undergoing different treatment options. Fourth, the analyses are largely ROI-specific. Fifth, no study have examined longitudinal treatment response outcomes. Lastly, many studies did not include a wait-list control group; only one study explored neural differences in treatment response between treatment types. For a pictorial overview of findings, see Fig. 2.

## Question 3: are there neuroimaging-based biotypes that define PTSD?

Psychiatry is moving towards a more precision-medicine approach, aiming to improve objective diagnosis, prediction, and treatment of mental disorders. Currently, to be diagnosed with PTSD, participants need to meet a certain number of symptoms that are largely self-reported and subjective, making the disorder highly heterogeneous [172]. To overcome the weak link between subjective-based diagnostic methods and objective-based neuroimaging assessments, recent studies have aimed to stratify PTSD to identify consistent subgroups based on objective brain-based markers [173–175]. Accordingly, Stevens and colleagues (2021) conducted a pioneering study to identify brain-based biotypes of psychiatric vulnerability shortly after trauma [176]. Using two cohorts from the AURORA longitudinal study of trauma survivors (n = 69 discovery cohort; n = 77 internal replication cohort) [112], the authors found and replicated three clusters based on early post-trauma brain activity during fMRI tasks assessing threat and reward reactivity, as well as response inhibition. These clusters were associated with distinct clinical trajectories up to 6 months post-trauma, with the group showing increased reactivity to threat and reward experiencing the most severe subsequent PTSD and anxiety symptoms [176]. In collaboration with Stevens and colleagues, Ben-Zion and colleagues (2023) conducted a conceptual replication of these brain-based biotypes [177] using a comparable dataset from the NMPTDT longitudinal study of trauma survivors [111]. While the authors found four clusters based on task-based fMRI data, they were not identical to the previously identified biotypes and were associated with prospective PTSD or anxiety symptoms. While there were many differences between the studies (AURORA and NMPTDT) that could contribute to the non-replication, this study highlights that additional replication studies are needed to identify more stable and generalizable neuroimaging-based biotypes before treatment implications can be fully realized [177, 178].

## Overall implications, future directions, and limitations

This narrative review aimed at exploring progress made in the past decade on three major questions in the field: (1) Which neural alterations serve as predisposing (pre-exposure) risk factors for PTSD development, and which are acquired (post-exposure)? (2) Which neural alterations can predict treatment outcomes and define clinical improvement? and (3) Can neuroimaging measures be used to define brain-based biotypes of PTSD? We present a synthesis of neuroimaging studies from the past decade in adults and youth with PTSD. Below, we present implications, provide areas of future research to be explored for each question, and highlight the limitations of our narrative review.

In the past decade, neuroimaging research on PTSD has advanced our understanding of the causal pathways of neural alterations within the disorder. However, we still cannot use the current neuroimaging knowledge to predict PTSD symptom trajectories or improve prevention and treatment options. Many of the above findings require replication in larger and more diverse samples with different trauma types across different methodologies. Importantly, future models used to determine the risk of developing trauma-related psychopathology will likely include information regarding demographics, socioeconomic status, and other clinical characteristics; thus, it is important to consider these factors when designing forthcoming studies. In the last ten years, we have seen a surge of longitudinal studies that collect neuroimaging measures early post-trauma and again at subsequent time points. While such studies are resource-intensive, scientists can answer questions not asked before, largely because of the development of large collaborations across multiple sites such as NMPTDT and AURORA research initiatives. The continuation and creation of more collaborations like these, with a focus on the collection of neuroimaging data shortly after trauma (and if possible, even pre-trauma) and at subsequent time points post-trauma, will be crucial in providing evidence to answer the vulnerability versus acquired characteristics of PTSD. To capture the dynamic evolution of the post-traumatic stress response, it is essential to incorporate multiple time points and ensure an adequately long follow-up period post-trauma (e.g., more than a year post-trauma), during which most of the recovery is anticipated.

Much work has been done to determine neural pre-treatment predictors of response and whether treatment normalizes alterations found in the disorder. While we have a relative understanding of potential predictors and changes associated with treatment response, much work still needs to be done to use this information in the clinic. Many of the studies reviewed had small sample sizes, used different treatment options, and samples were not diverse regarding sociodemographic factors and trauma type. Future studies should seek to replicate previous findings with bigger sample sizes, comparisons should be made between treatments, and more community-based samples should be prioritized.

The significant clinical heterogeneity observed in PTSD (and other post-traumatic psychopathologies), coupled with recent advancements in statistical and computational techniques, has spurred the pursuit of identifying homogeneous PTSD subtypes using data-driven methodologies. However, the assumption of distinct and homogeneous subgroups may not be clinically useful or accurately reflect the underlying biology of PTSD. For instance, most clustering methods will invariably produce clusters, even without any inherent data structure, highlighting the importance of differentiating between biologically and clinically relevant subtypes and random data fluctuations or noise [179, 180]. Future research aiming to identify brain-based biotypes of PTSD will benefit from global collaborations between research teams, combining unique large-scale datasets and sharing of analytic pipelines (as exemplified recently by Stevens [176] and Ben-Zion [177]). Furthermore, subsequent studies will benefit from employing hybrid methodologies that integrate theory- and data-driven approaches Field [195,196] and implementing open science protocols (e.g., preregistration, transparent reporting of all results).

Few investigations explored here explicitly examined sex as a biological variable. As has been recently reviewed [13], the underlying neurobiological correlates of sex differences in PTSD are unknown. Most of the studies discussed here did not include a direct comparison between males and females. However, a handful of studies did examine female-only or male-only samples, which do not allow for the generalization of findings to the opposite sex. Thus, more studies should examine sex differences in their samples.

We have highlighted findings in youth. Still, much more work is needed to parse better the brain’s natural development versus the impact trauma may have on brain regions. Few studies have explored the questions posed in youth samples. Though resources are a limiting factor, future studies should execute longitudinal study designs that start in youth to determine the role of childhood trauma, potentially before it happens, in the development of trauma-related psychopathology in adulthood. Additionally, genetic and neurobiological studies linking transgenerational PTSD presentation would be beneficial in parsing preventative markers for developing PTSD. Thus, focusing on youth populations would be optimal in answering our first main question.

### Limitations

There are notable strengths of this narrative review, including providing a synthesis of neuroimaging studies in both adult and youth samples that explore three leading questions in the PTSD field. Despite these strengths, limitations do exist. First, the authors have tried to include all the pertinent studies to answer the three questions, though a systematic protocol was not used when exploring studies. Second, given the number of studies available to be reviewed, the conclusions drawn from each question are limited. Further, as outlined above, methodological variability exists in the studies reviewed, including differences in scanning parameters and PTSD samples. This variability limits the reliability and validity of the conclusions made. Regardless of these limitations, this review is important as it provides insight into where the field stands on these three questions, highlighting that much research still needs to be conducted to make stronger conclusions.

Despite the limitations of the studies reviewed and of this narrative review, PTSD neuroimagers have made much progress in the last decade and have much more to make, especially in answering questions related to disparities in the development of the disorder and translating the knowledge collected beyond academia, to the communities we serve.
